# The EPH/Ephrin System in Bone and Soft Tissue Sarcomas’ Pathogenesis and Therapy: New Advancements and a Literature Review

**DOI:** 10.3390/ijms23095171

**Published:** 2022-05-05

**Authors:** Argyris C. Hadjimichael, Alexandros Pergaris, Angelos Kaspiris, Athanasios F. Foukas, Stefania Kokkali, Gerasimos Tsourouflis, Stamatios Theocharis

**Affiliations:** 1 First Department of Pathology, Medical School, National and Kapodistrian University of Athens, 75 Mikras Asias Street, 11527 Athens, Greece; ortho.argiris@gmail.com (A.C.H.); alexperg@yahoo.com (A.P.); stefkokka@med.uoa.gr (S.K.); gtsourouflis@med.uoa.gr (G.T.); 2 Department of Orthopaedics, St Mary’s Hospital, Imperial College Healthcare NHS Trust, Praed Street, London W2 1NY, UK; 3 Laboratory of Molecular Pharmacology, Department of Pharmacy, School of Health Sciences, University of Patras, 26504 Patras, Greece; angkaspiris@hotmail.com; 4 Third Department of Orthopaedic Surgery, “KAT” General Hospital of Athens, Nikis 2, 14561 Kifissia, Greece; afoukas1@otent.gr

**Keywords:** EPH, ephrin, bone sarcoma, soft tissue sarcoma, in vitro, in vivo

## Abstract

Musculoskeletal sarcomas represent rare heterogenous malignancies of mesenchymal origin that can be divided in two distinct subtypes, bone and soft tissue sarcomas. Current treatment options combine the surgical excision of local tumors and multidrug chemotherapy to prevent metastatic widespread disease. Due to the grim prognosis that usually accompanies such tumors, researchers have attempted to shed light on the molecular pathways implicated in their pathogenesis in order to develop novel, innovative, personalized therapeutic strategies. Erythropoietin-producing human hepatocellular receptors (EPHs) are tyrosine-kinase transmembrane receptors that, along with their ligands, ephrins, participate in both tumor-suppressive or tumor-promoting signaling pathways in bone and soft tissue sarcomas. The EPH/ephrin axis orchestrates cancerous processes such as cell–cell and cell–substrate adhesion and enhances the remodeling of the intracellular cytoskeleton to stimulate the motility and invasiveness of sarcoma cells. The purpose of our study was to review published PubMed literature to extract results from in vitro, in vivo and clinical trials indicative of the role of EPH/ephrin signaling in bone and soft tissue sarcomas. Based on these reports, significant interactions between the EPH/ephrin signaling pathway and a plethora of normal and abnormal cascades contribute to molecular mechanisms enhancing malignancy during sarcoma progression. In addition, EPHs and ephrins are prospective candidates for diagnostic, monitoring and therapeutic purposes in the clinical setting against bone and soft tissue sarcomas.

## 1. Introduction

Bone and soft tissue sarcomas represent a family of rare connective tissue malignancies with mesenchymal origin and very aggressive behavior [1,2,3]. Based on epidemiological reports from EUROCARE (European Cancer Registry based study on survival and care of cancer patients), primary bone sarcomas account for less than 0.2% of malignant neoplasms [4]. Meanwhile, the incidence of soft tissue sarcomas in Europe is approximately 3.6–4.7 cases per 100,000 people annually, representing less than 1% of all cancer types [5]. The most common histological types of bone sarcomas are osteosarcoma, Ewing’s sarcoma and chondrosarcoma [6], whereas rhabdomyosarcoma, synovial sarcoma, undifferentiated pleomorphic sarcoma and leiomyosarcoma are the most usually diagnosed histological types for soft tissue sarcomas [6].

Regarding prognosis of bone sarcomas, the age-standardized relative survival reduces to 78% at one year, 60% at three years and 53% at five years after diagnosis [7]. Similarly, the age-standardized relative survival for all ages is deteriorated to 82% at one-year, 66% at three years and 60% at five years after a soft tissue sarcoma is confirmed. The American Joint Committee on Cancer (AJCC) correlates the poorer prognosis with predictors such as the local expansion of tumor and the presence of metastatic disease in lymph nodes and distant sites (e.g., lungs, liver, bones) [8]. Oftentimes, localized sarcomas can be successfully treated with surgery and radiation, but metastatic widespread disease can be prevented only by chemotherapy [9]. The necessity for the development of novel anti-metastatic drugs has emerged in recent years, prompting the investigation of metastatic pathways and patterns [10].

**Figure 1:**
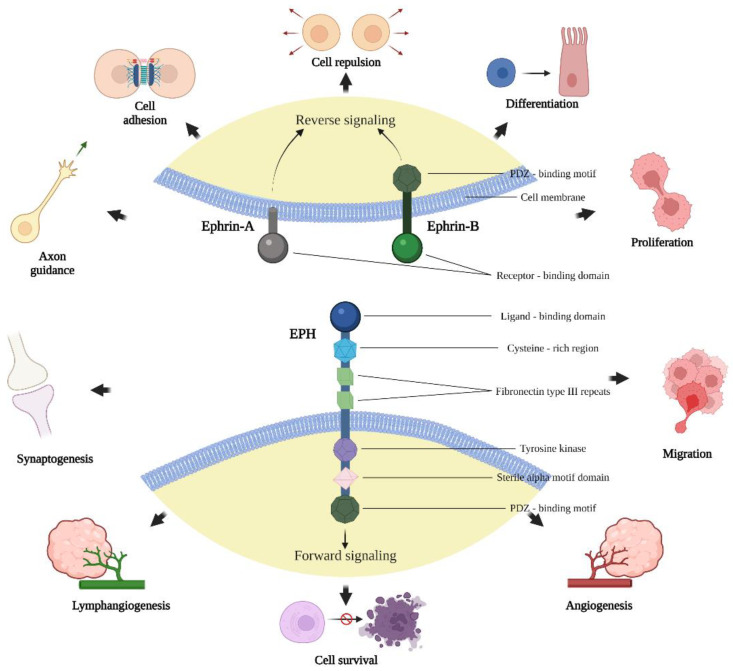
Structure of EPHs and ephrins and the presentation of some physiologic processes mediated through the EPH/ephrin signaling pathway. Created with BioRender.com, accessed on 2 May 2022.

Erythropoietin-producing human hepatocellular receptors (EPHs) is a large family of membrane-bound tyrosine kinases receptors (RTKs) which bind the Eph family receptor interacting proteins (ephrins) located on the surfaces of neighboring cells [11]. EPHs are further divided in two subfamilies. The first family consists of type-A EPHs (EPHA1–EPHA8 and EPHA10), which interact with type-A ephrins (ephrin-A1–ephrin-A5) [12]. The second family consists of type-B, EPHs (EPHB1–EPHB4 and EPHB6), which interact with type-B ephrins (ephrin-B1–ephrin-B3). However, cross-interaction between members of the two categories has been described [12]. For instance, EPHB2 can activate ephrin-A5, and EPHA4 can activate ephrin-B ligands. Likewise, the interaction and activation between EPHs and multiple ephrins can be seen within categories (e.g., EPHB2 activates ephrin-B1, B2 and B3) [12]. The EPH/ephrin signaling and cell–cell interactions regulate many physiologic and homeostatic events (Figure 1). For example, one of the roles of the EPH/ephrin system is to regulate neurogenesis and neuronal migration during embryogenesis and to guide axonal growth during early brain development [13]. Furthermore, it has been suggested that EPHA/ephrin-A signaling enhances osteoclastogenesis and suppresses osteoblastogenesis in vitro [13]. Additionally, it has been found that ephrin-B reverse signaling inhibits osteoclast differentiation and deteriorates bone resorption, whereas EPHB promotes the differentiation of osteoblasts, leading to increased bone formation [13]. Therefore, the EPH/ephrin system may function as an important regulator in normal bone homeostasis. Moreover, there is strong evidence that the interaction between EPHB4 and ephrin-B2 contributes to the formation of new blood vessels (angiogenesis) and lymphatic vasculature (lymphangiogenesis) [13]. This is one of the most significant mechanisms that malignant tumors use to achieve rapid growth and distant metastatic dissemination. An effective strategy to inhibit the progression of primary tumors and the expansion of lethal metastatic sites is to block the process of neo-angiogenesis that supports tumor cells with oxygen and adequate blood supply. Clearly, targeted anti-EPH/ephrin agents could influence the aggressive potential of bone and soft tissue sarcomas and open up the possibility to generate novel chemotherapeutic drugs.

In addition, the EPH/ephrin system is involved in malignant processes such as metastatic development [13]. Subsequently, several in vitro and in vivo studies have elucidated the molecular mechanisms through which the EPH/ephrin signaling pathway has impact on tumorigenesis and cancer metastasis [14]. For example, the EPHB4/ephrin-B2 interaction enhances angiogenesis via the VEGFR2 and Notch signaling pathways in glioblastoma [14]. In addition, the phosphorylation of EPHA3 activates the PI3K/Akt and MAPK pathways and induces resistance to trastuzumab in breast cancer [14]. On the contrary, EPHA3 activates the phosphorylation of the PI3K/BMX/STAT3 signaling pathway, leading to the apoptosis of lung carcinoma cells [14].

The aim of our review is to summarize and present the data from studies that attempted to evaluate the relationship between the EPH/ephrin signaling pathway and the progression of bone and soft tissue sarcomas. Moreover, we will explore the role of EPHs and their ligands as biomarker candidates, prognostic and monitoring tools and potential specialized therapeutic targets in bone and soft tissue sarcomas.

## 2. Methodology

### 2.1. Research Strategy

The authors investigated published studies addressing the attributes of the EPH/ephrin signaling pathway in bone and soft tissue sarcomas. In addition, we investigated the role of EPHs and ephrins as biomarkers, prognostic and monitoring molecules as well as therapeutic targets against bone and soft tissue sarcomas. A systematic computer-based literature review search along with predefined criteria was performed in MEDLINE/PubMed (1946 until present) of the National Library of Medicine and in EMBASE (1947 until present). The combination of the following terms was used: “EPH/ephrin (All fields)”, “bone sarcomas (All fields)”, “soft tissue sarcomas (All fields)”, “in vivo (All fields)”, “in vitro (All fields)”, “diagnosis (All fields)”, “monitoring (All fields)”, “prognosis (All fields)”, “treatment (All fields)”. The electronic literature search was performed by two authors independently (A.C.H., A.P.). In addition, the senior authors (A.K., A.F.F., S.K., G.T. and S.T.) screened the titles and abstracts independently to detect relevant studies investigating the role of EPH/ephrin signaling in bone and soft tissue sarcomas. Eventually, based on clinical and laboratory findings, the EPHs/ephrins gene and protein expression were correlated to pathological parameters, including histological grade, disease stage, presence of lymph node or distant metastasis especially those in lungs, prognosis, overall and disease-free survival.

### 2.2. Inclusion and Exclusion Criteria

Published articles written in English, peer-reviewed journals and clinical studies concerning the impact of the EPH/ephrin signaling pathway in bone and soft tissue sarcomas were considered. Likewise, in vitro and in vivo experimental studies were included.

However, articles written in language other than English, letters to the editor, expert opinion publications and surveys with insufficient and inappropriate details on the impact of Eph/ephrin signaling pathway in bone and soft tissue sarcomas were excluded. Additionally, published articles exhibiting the impact of EPHs and ephrins in Kaposi’s sarcoma were not considered. The Endnote software (Clarivate Analytics, Philadelphia, PA, USA) was used to assess the presence of duplicate studies, which were eventually excluded.

### 2.3. Data Extraction

Published data from articles that were in agreement with the previously mentioned criteria were collected and were extensively investigated. Three senior authors (A.K., A.F.F. and S.T.) examined all appropriate surveys and extracted data. Two authors (A.C.H., A.P.) extracted data from reviewed studies about the impact of EPH/ephrin system in bone and soft tissue sarcomas, and results are presented in the article’s figures.

## 3. EPH/Ephrin Signaling Pathways in Bone Sarcomas

### 3.1. EPH/Ephrin Expression in Osteosarcoma and Interaction with Ras/MAPK Pathway

The experimental study by Fritsche-Guenther et al. revealed that EPHs/ephrins are either being upregulated or de novo expressed during the oncogenic signaling of osteosarcoma, stimulating its metastatic phenotype [15]. According to their findings, the expression of ephrin-A1, a ligand with high affinity with the EPHA tyrosine kinase receptors, was found 10-fold higher in osteosarcoma cells compared to normal osteoblasts. Furthermore, EPHA2 was present only in osteosarcoma samples but absent in non-malignant bone cells [15]. In addition, ephrin-B1 levels were found de novo increased in primary osteosarcoma tumors but were significantly downregulated in metastatic osteosarcoma of the lungs [15]. Likewise, the expression of ephrin-B3 and EPHA3 were found elevated in osteosarcoma without a significant difference being reported between primary and metastatic tissue samples [15]. The interaction between EPHA2 and ephrin-A1 enhanced the phosphorylation of EPHA2′s tyrosine and increased the mitogenic process via the Ras/MAPK pathway, which eventually enhanced the proliferation and migration of SaOS2 and MNNG/HOS human osteosarcoma cells [15].

### 3.2. EPHA2/Ephrin-A1–CAV1 Axis Activates AKT Signaling in EWS

The impact of EPH/ephrin signaling in neo-angiogenesis and the tumor neovascularization process was correlated with the highly aggressive behavior of Ewing’s sarcoma by Sáinz-Jaspeado et al. [16]. Their in vitro trial demonstrated that the silencing of the metastasis-associated *CAV1* gene, which expresses the CAV1 integral protein, was related to reduced endothelial cell migration in Ewing’s sarcoma [16]. According to their findings, the interaction between CAV1 and EPHA2 in the presence of ephrin-A1 activates the AKT signaling pathway and promotes the expression of the pre-angiogenic basic fibroblast growth factor (bFGF). Subsequently, the secreted bFGF acts as a chemoattractant for endothelial cells and promoter of angiogenesis in Ewing’s sarcoma [16]. Likewise, their in vitro findings were in line with in vivo outcomes after the implantation of three Ewing’s sarcoma cells with the *CAV1* gene silencing profile and low expression of CAV1 transmembrane protein. The primary tumor growth, along with the number of vessels supplying it, were found significantly reduced, leading to excessive tumor necrosis. The EPHA2/ephrin-A1–CAV1 axis activates AKT signaling to secrete bFGF and has been proposed as a potential target of chemotherapeutic agents [16].

### 3.3. Activation of Notch Signaling Pathway by EphrinB1 in Osteosarcoma

Another study by Yu et al. provided pre-clinical and clinical in vitro evidence that the interaction between Notch signaling and the EPH/ephrin axis contributes to osteosarcoma progression [17]. The Notch signaling pathway consists of four Notch receptors (Notch 1–4), which are single-pass type I transmembrane molecules, and their transmembrane ligands Delta-like and Jagged [18]. Upon ligand activation, the Notch intracellular domain (NICD) is translocated to the nucleus to activate the transcriptional cofactor CBF1, leading to the expression of *HES* and *HEY* genes [18]. In vitro, the mRNA expression levels of Hes1 from the Notch pathway and ephrin-B1 from the EPH/ephrin axis were found to be both elevated in 143B highly metastatic human osteosarcoma cells. Further in silico analysis revealed that −1438 to −1431, −2430 to −2423, and −2911 to −2904 in the ephrin-B1 promoter region are the three recognized binding sites for NICD1 [17]. The activation of the Notch pathway promotes the phosphorylation and overexpression of ephrin-B1 [17]. Based on the experimental observations by Yu, eprin-B1 enhances the Notch-driven osteosarcoma cells’ proliferation and chemoresistance, properties which were successfully reversed in ephrin-B1 knockdown in the aforementioned cells [17]. Eventually, specific anti-ephrin-B1 agents represent potential drug candidates that could eliminate the expansion and metastatic burden of osteosarcoma as well as counter the cells’ chemoresistance toward existing chemotherapeutic regiments.

### 3.4. The Key Role of EPHA2 in Osteosarcoma, Chondrosarcoma, Ewing’s Sarcoma

Among others, one of the synergistic antitumor activities that pazopanib and trametinib (receptor tyrosine kinase inhibitors) exhibit in osteosarcoma cells is carried out through the down-modulation of EPHA2 and Interleukin (IL)-7 Receptor (IL-7R), according to the in vitro findings by Chiabotto et al. [19]. Initially, a significant suppression of migration and proliferation properties was observed in four EPHA2-silenced osteosarcoma cell lines (HOS, KHOS/NP, MNNG/HOS and U2OS) compared to their controls. Importantly, for the first time, the combination of drugs such as pazopanib and trametinib has been proposed as an EPHA2 oncogene suppressor with potential therapeutic effect in the clinical setting for unresectable or metastatic osteosarcoma cases [19].

The potential role of EPHA2 as a monitoring molecule and pharmacological target in osteosarcoma, Ewing’s sarcoma and chondrosarcoma was interpreted by a study that was conducted by Giordano et al. in 2021 [20]. Using bioinformatic analysis, the EPHA2 expression levels were correlated to patients’ clinical outcomes according to TARGET-OS project data extracted from the NCI Genomic Data Commons (88 osteosarcoma patients) as well as from three public gene expression experiments deposited in the Gene Expression Omnibus (90 osteosarcoma patients) [20]. The authors confirmed in silico that the expression of EPHA2 in samples retrieved from patients with Ewing’s sarcoma was higher compared to normal tissues [20]. In addition, higher measured levels of EPHA2 were indicative of advanced Huvos grade in osteoblastic osteosarcoma and poorer prognosis in patients with dedifferentiated chondrosarcoma. Likewise, the expression levels of EPHA2 were found to be significantly higher among male compared to female Ewing’s sarcoma and osteosarcoma patients, which was in accordance with the prognostic value of gender for those bone malignancies [20]. Another interesting data extracted from this study was that the phosphorylation of EPHA2 at its critical serine 897 contributes to the activation of the oncogenic non-canonical process in both osteosarcoma and chondrosarcoma patient-derived xenograft models. The generation of *p*-EPHA2*^Ser897^* might be responsible for the evolution to a more aggressive phenotype and to metastatic disease. This finding was not confirmed in Ewing’s sarcoma patient-derived xenografts due to the variability of expression levels for *p*-EPHA2*^Ser897^* in this type of sarcoma [20]. Finally, the potent EPHA2 inhibitor ALW-II-41-27 demonstrated remarkable impeding effects on cell growth and cell viability when applied in osteoblastic osteosarcoma, Ewing’s sarcoma and conventional chondrosarcoma [20]. Despite the association between EPHs/ephrins with tumorigenesis and cancer progression, a study by Kalinski et al. revealed that ephrin-A5 has a protective role in chondrosarcoma pathogenesis [21]. Results from their in vitro analysis showed a significant downregulation of ephrin-A5 at the transcriptional level in chondrosarcoma cells compared to normal ones. Therefore, ephrin-A5 is not implicated in cell–cell adhesion interactions that could remodel the microenvironment to promote the expansion of chondrosarcoma. Thus, ephrin-A5 could have been investigated as a potential tumor-suppressing ligand through the interaction with its tumor promoting receptors such as EPHA2, EPHA3, EPHA4, EPHA5, EPHA7, EPHA8, and EPHB2 in sarcomas [21].

### 3.5. The Role of EPHA7 in HCP5/miR-101/EPHA7 Axis in Osteosarcoma

A novel mechanism involving the expression of EPHA7 in the progression of osteosarcoma has been recently proposed by Tu et al. in 2021 [22]. EPHA7 has the ability to interact with HCP5 (long non-coding RNAs (lncRNA) of human histocompatibility leukocyte antigen (HLA) complex P5) and miR-101, a non-coding microRNA [22]. In the clinical setting, a high expression of HCP5 in tissue samples retrieved from osteosarcoma patients was significantly correlated to low survival and poor prognosis. On the contrary, the in vitro downregulation of HCP5 led to a notable inhibition of proliferation, migration, invasion and enhanced apoptosis in osteosarcoma cell lines [22]. The experimental data of this study revealed that HCP5 directly targets and regulates the expression levels of miR-101. Likewise, the miR-101 directly targets the EPHA7 (the binding site of miR-101 is EPHA7 3′UTR), regulating its expression. Therefore, the HCP5/miR-101/EPHA7 axis has been correlated to osteosarcoma malignant development, as HCP5 promotes the increased expression of EPHA7 via targeting miR-101 competitively [22]. Consequently, HCP5, miR-101 and EPHA7 could be further considered as potential prognostic biomarkers and therapeutic targets for osteosarcoma treatment.

### 3.6. Ephrin-Specific Expression Profile in Osteosarcoma

Varelias et al. revealed that a specific ephrin profile is present in human osteosarcoma specimens and human osteosarcoma cell lines, which is correlated with the progression of malignancy [23]. According to their findings, two mRNA profile patterns were recognized between normal bone tissues, osteosarcoma samples and osteosarcoma cells. The first mRNA profile included the expression of ephrin-A1, ephrin-A4 and ephrin-B2, which coordinate migration and cell–cell contact, oftentimes being involved in osteoblasts regulating bone homeostasis [23]. The second mRNA profile was composed of ephrin-A3, ephrin-A5, and ephrin-B1 in a subset of osteosarcoma patients with possibly worse prognosis [23]. The most significant observation was that increased levels of ephrin-B1 were detected in osteosarcoma cells and blood vessels and were associated with local recurrence, metastatic disease and poorer clinical prognosis [23]. Therefore, the interaction between B-subclass ephrins and EPHs may influence patients’ prognosis via excessive tumor neovascularization, which promotes metastatic spread through newly formed blood vessels and assists the construction of tumor vascular networks for nutritional and oxygen supplies at metastatic sites [23].

**Figure 2:**
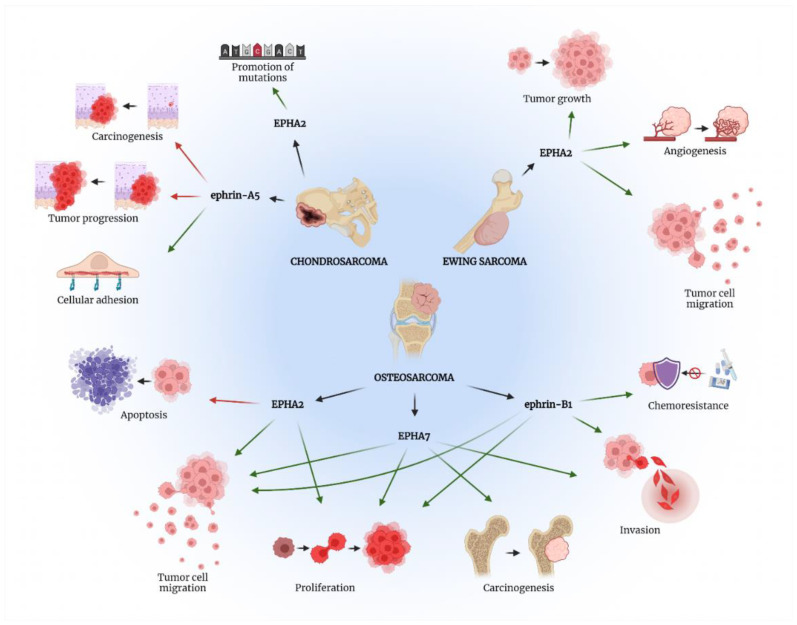
The role of EPH/ephrin axis in bone sarcomas’ pathogenesis. Green arrows present procedures promoted by the specific EPH/ephrin member, while red arrows show processes suppressed by the aforementioned molecules. Created with BioRender.com, accessed on 2 May 2022.

The EPHs/ephrins investigated in bone sarcomas’ pathogenesis as well as the results reported are summarized in Table 1 and Figure 2.

## 4. EPH/Ephrin Signaling Pathways in Soft Tissue Sarcomas

### 4.1. The EPH/Ephrin Pathway in SYT-SSX2 Positive Synovial Sarcoma

Synovial sarcoma represents a unique soft tissue cancer accounting for up to 5–10% of all soft tissue sarcomas and affecting mostly young populations [24]. Notably, synovial sarcoma is associated with the genetic translocation t(X:18), which generates either SYT-SSX1 or SYT-SSX2 tumor-promoting proteins [24]. Synovial sarcoma cells transduced with the SYT-SSX2 carcinogenic genotype often reveal a unique cytoskeletal phenotype due to the direct activation of the EPH/ephrin signaling pathway [25]. The in vitro trial by Barco et al. showed that the SYT-SSX2 oncogene acts as a “positional mediator” rather than a “proliferative mediator” through its ability to alter the morphology of tumor cells, rather than their ability for numerical expansion [25]. According to their findings, SYT-SSX2 induces the hyperphosphorylation of EPHB2 after interaction with ephrin-B1, resulting in the alteration of the cytoskeletal structure and its microtubular composition. Consequently, an EPHB2-mediated cytoskeletal remodeling is observed, which is characterized by the elongation and narrowing of synovial sarcoma cells, producing neurite-like extensions [25]. Hence, the activation of the EPH/ephrin pathway appears as a potential in vivo mechanism that promotes a repulsive effect responsible for the loss of cell–cell adhesion and stimulation of the metastatic cascade in synovial sarcoma.

### 4.2. EPHs and Ephrins Enhance the Metastatic Potential in Rhabdomyosarcoma

Evidence from published studies suggest the important role of EPH/ephrin signaling in the progression of rhabdomyosarcoma, which represents a highly malignant and a fast-growing soft tissue tumor. Rhabdomyosarcoma is the most commonly observed type of soft tissue sarcoma in children and adolescents [26]. Embryonal rhabdomyosarcoma (ERMS), alveolar rhabdomyosarcoma (ARMS) and spindle cell-sclerosing rhabdomyosarcoma (SRMS) comprise the three major histological subtypes, which arise from mesenchymal progenitor cells due to developmental disruptions instead of being differentiated into striated muscle cells [26]. Initially, a dysregulation of mRNA expression levels for EPHs and ephrins was noted by Berardi et al. in rhabdomyosarcoma cell lines compared to normal muscle cells [27]. In ERMS primary tumors and cell lines, the overexpression of ephrin-B1 was associated with increased measured levels of EPHB1 and EPHB3. Similarly, the overexpression of ephrin-B2 was correlated with the overexpression of EPHB1, EPHB2 and EPHB4 for the same histological subtype [27]. However, no specific correlations were found between EPHs and ephrins in ARMS tumors [27]. Overall, the global deregulation of EPHs and ephrins could justify metastatic features in rhabdomyosarcoma, such as increased motility, due to cell–cell detachment and higher invasiveness [27].

### 4.3. Tumor-Suppressive Effect of EPHA3 in Rhabdomyosarcoma

Although many studies have indicated the tumorigenic effect of the EPH/ephrin signaling in sarcomas, a study by Clifford et al. has illustrated the protective role of EPHA3 against rhabdomyosarcoma progression [28]. According to their in vitro trials, the EPHA3 expression was suppressed in rhabdomyosarcoma cell lines that harbor chromosomal translocations, which are associated with enhanced aggressiveness and metastatic potential [28]. For instance, the upregulation of EPHA3 was present in two ERMS (TE671 and RD) cell lines and one ARMS (FLOH-1) cell lines that do not express the PAX3-FKHR fused oncogene. On the contrary, the downregulation of EPHA3 was found in highly aggressive ARMS cell lines (CRL2061 and KM77) that exhibit the *PAX3-FKHR* gene translocation [28]. It has been observed that enhanced interaction between EPHA3 and its ligand ephrin-A5/Fc in rhabdomyosarcoma modulates Rho GTPases, leading to a decrease in cell–fibronectin adhesion and migration toward stromal cell-derived factor 1 (SDF-1) [28]. According to Clifford et al., the phosphorylation of the following three tyrosines within EPHA3 is responsible for its fundamental enzymatic activity: two conserved tyrosines in the juxta membrane region that are mutated in phenylalanine and a third tyrosine in the activation loop, which is also mutated to phenylalanine [28]. These data indicate that the activation of EPHA3 with ephrin-A5 pharmaceutical analogues could be a future chemotherapeutic strategy to suppress the motile and metastatic phenotype of tumor cells in rhabdomyosarcoma patients.

### 4.4. Cross Interaction between EPH/Ephrin Pathway and PDGFRβ/PDGFR-BB Axis

One of the previous attempts for the detection of novel therapeutic targets during the progression of the highly aggressive ARMS has been conducted by Aslam et al. [29]. According to the researchers’ findings, the cross-reaction between EPHB4 and platelet-derived growth factor receptor-β (PDGFRβ), which also constitutes a tyrosine kinase receptor, contributes to the excessively poor prognosis in ARMS patients [29]. Clinicopathologic correlations have identified a significant association between PDGFRβ signaling in vascular stroma and metastatic status of ARMS tumors [30]. The in vitro analysis of the aforementioned research team in murine and human ARMS tissues revealed that the phosphorylation of EPHB4 is induced by PDGFRβ when the latest is being stimulated by its ligand, PDGF-BB [29]. Subsequently, the PDGF-BB ligand promotes the direct activation of PDGFRβ and indirect activation of EPHB4, which eventually stimulates Akt and Erk1/2 signaling pathways that enhance tumor cell survival and proliferation, respectively [29]. Further application of dasatinib, a non-selective FDA-approved tyrosine kinase inhibitor, in cultured cells as well as in orthotopically engrafted xenograft models, reduced tumor expansion in vitro and prolonged the survival of ARMS xenografts in vivo [29]. Therefore, the antagonistic effect of dasatinib against PDGFRβ and EPHB4 could possess a therapeutic antitumor perspective in ARMS.

### 4.5. EPHA3/Ephrin-A1 Pathway as a Mediator for NCAM in PAX Positive ERMS

ERMS is the most frequent RMS histological variant, as it accounts for up to 60–70% of all diagnosed RMS tumors, with no gene translocations being recognized until recently [31]. However, the overexpression of the transcriptional factor PAX7 remains a hallmark of ERMS tumors, due to its ability to regulate cancerous properties such as cell adhesion, migration and invasiveness [31]. In vitro as well as in vivo, PAX7 boosted the upregulation of EPHA3 and its ligand, ephrin-A1, and it downregulated the neural cell adhesion molecule (NCAM1) in ERMS cells. As a result, ERMS cells preserved decreased levels of NCAM/polysialylated–NCAM ratio and exhibited the excessive migration and invasive properties of the PAX7-overexpressing cells [31]. Interestingly, the ectopic upregulation of the receptor for advanced glycation end-products (RAGE) activates a RAGE/myogenic axis that reverses the EPHA3/ephrin-A1 pathway and downregulates PAX7 [31]. Thus, EPHA3 and ephrin-A1 appear as intermediate regulators and potential therapeutic ERMS suppressors.

### 4.6. Interaction between Ephrin-B1 and Histone Deacetylases in ERMS

Histone deacetylases (HDACs) consist of a family of enzymes that act by removing acetyl groups from the NH2 terminal tails of DNA-binding histone proteins, thus decreasing the accessibility of chromatin for transcription factors [32,33,34]. Therefore, HDACs are able to modify molecular mechanisms, such as post-translational or epigenetic regulation, which play a significant role in the pathogenesis of ERMS [35]. In a study by Vleeshouwer-Neumann et al., the in vitro administration of trichostatin A (TSA) or suberoylanilide hydroxamic acid (SAHA, also known as vorinostat), which represent HDAC inhibitors in ERMS cells, achieved a reduction in the expression of ephrin-B1 through the binding of these inhibitors on the *ephrin-B1* promoter region [35]. As a result, the migratory capacity of ERMS cells was reported as suppressed, but this effect was not observed in the progression of tumor cells’ abnormal cell cycle as well as in differentiation [35]. In addition, ephrin-B1 is significantly overexpressed in ERMS patients compared to ARMS ones [35]. Consequently, ERMS patients could strongly benefit from treatment with HDAC inhibitors, as they exhibit potential anti-metastatic properties by reducing the migratory behavior of ERMS cells through ephrin-B1 inhibition.

### 4.7. Inhibition of the EPHB4/Ephrin-B2 Pathway in ARMS and ERMS

Recently conducted research has investigated the EPH/ephrin pathway for the design of molecularly targeted therapies and novel chemotherapeutic agents to control local tumors and prevent metastatic disease in rhabdomyosarcoma. Randolph et al. utilized in vivo allograft and xenograft preclinical mouse models to evaluate two EPHB4 inhibitors: the VasG3 antibody and the serum EPHB4-HSA (human serum albumin) fusion protein against ARMS and ERMS [36]. According to their reports, the VasG3 treatment did not deteriorate tumor progression, and sEPHB4-HAS slightly decreased tumor growth in ARMS murine models. Likewise, neither VasG3 nor sEPHB4-HAS demonstrated better survival rates or reduced tumor growth rates in human ERMS xenografts mice and ERMS patient-derived preclinical models [36]. Taking data together, VasG3 and sEPHB4-HAS could not be used as monotherapy treatments by inhibiting the single molecular pathway EPHB4/ephrin-B2. Instead, a more sufficient tumor suppressing effect might be achieved by inhibiting multiple kinase targets and pathways, as shown by Aslam et al., using dasatinib (a broad spectrum, non-selective tyrosine kinase inhibitor) [29].

### 4.8. Inhibition of EPHA2 and EPHBs Blocks the AKT/mTOR and MEK/ERK Pathways in ERMS

The pharmacological inhibition of the EPH/ephrin signaling pathway in ERMS was proposed by Megiorni et al., who reported the significant antitumor effects of GLPG1790, a novel potent pan-EPH inhibitor [37]. Treatment with GLPG1790 decreased the in vitro phosphorylation/activation of EPHA2 and EPHBs and eventually blocked the activation of the AKT/mTOR and MEK/ERK axis. Subsequently, GLPG1790 inhibited proliferation and reversed the malignant phenotype of ERMS cells toward normal skeletal muscle differentiation (increased expression levels of MYOD1, Myogenin and MyHC) [37]. Meanwhile, inhibition of the EPH/ephrin pathway by GLPG1790 enhanced JNKs-mediated apoptosis, occluded p-38 sustained differentiation and deteriorated motility and invasion features by restricting SRC-mediated integrin signals [37]. In addition, GLPG1790 amplified the radiosensitivity of ERMS cells via an increased impairment of the DNA double-strand break repair. In vivo, the combination of GLPG1790 and radiation therapy achieved an up to 83% reduction in primary tumor growth in ERMS xenograft models [37].

### 4.9. Inhibition of Tumor-Promoting EPHB4/Ephrin-B2 Axis by CAR-T Cells in ARMS and OS

An alternative pharmaceutical approach based on the immune response was designed by Kubo et al. in 2021, who developed novel chimeric antigen receptor (CAR)-T cells to target EPHB4 via their interaction with ephrin-B2 [38]. EPHB4-CAR-T cells were created via *piggyBac (PB)* transposon-based gene transfer and exhibited antitumor efficacy against EPHB4-positive rhabdomyosarcoma tumors. Engineered EPHB4-CAR-T cells showed sustained killing activity against osteosarcoma as well as against rhabdomyosarcoma cell lines, even for PAX3-FOXO1-positive ARMS cells, which exhibit highly malignant immunomodulatory effects [38]. In addition, the Ephrin-B2-Fc/EPHB4 interaction reduced the in vitro proliferation of rhabdomyosarcoma cells, prolonged the in vivo survival of rhabdomyosarcoma xenografts mice, and suppressed the growth rates of their primary tumors after receiving EPHB4-CAR-T cells compared to CD19-CAR-T cells (control) therapy [38].

**Figure 3:**
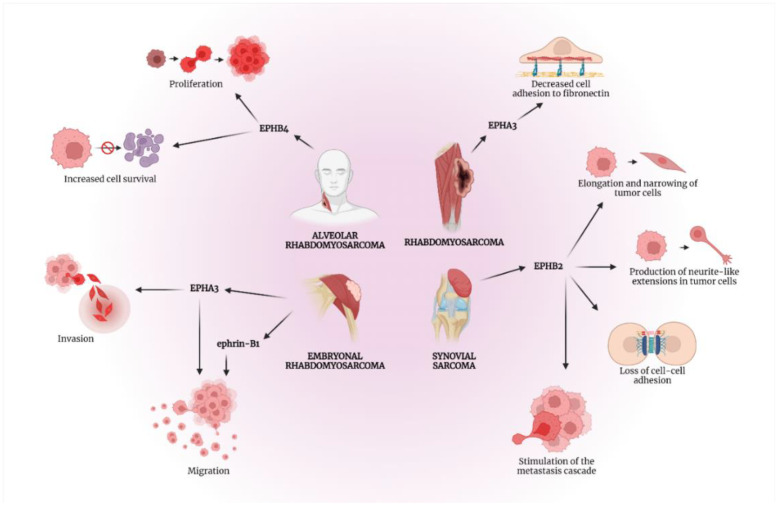
Various EPHs/ephrins are implicated in a multitude of tumor-promoting cellular processes in soft tissue sarcomas. Created with BioRender.com, accessed on 2 May 2022.

Table 2 and Figure 3 present the roles of various EPHs/ephrins in soft tissue sarcomas’ tumorigenesis.

## 5. The EPH/Ephrin System in the Treatment of Musculoskeletal Malignancies

Bone and soft tissue sarcomas are fast growing and difficult to cure musculoskeletal malignancies despite the establishment of intensive chemotherapy protocols and extensive surgical removal of primary tumors [39]. The contribution of the EPH/ephrin signaling pathway and its interactions in tumorigenesis gained significant attention in basic research in order to further unravel the mechanisms of progression and metastatic processes in bone and soft tissue sarcomas.

According to the existing literature, the EPH/ephrin signaling pathway coordinates a multitude of physiological processes such as embryogenesis, normal neurogenesis, blood and lymphatic vessels development as well as bone homeostasis [40]. However, multiple studies have proven the correlation between activation of the EPH/ephrin axis and tumorigenesis, with a remarkable impact on cancer stage, histologic grade and patient’s overall survival, as recently reviewed by our group [14]. Recently, a significant interest has risen regarding the involvement of EPHs and ephrins in oncogenesis, which oftentimes exhibit either tumor-suppressing or tumor-promoting roles [41,42]. As reviewed previously, the bidirectional signaling between the transmembrane and intracellular domains of EPHs and a plethora of ephrin subtypes modulates the activation of plenty other signaling pathways via cross-talk [43]. Consequently, the EPH/ephrin axis regulates the differentiation, proliferation and survival of tumor cells and remodels the tumor’s microenvironment by interfering with cytoskeletal signaling that has an impact on cell–cell adhesion and cell migration [43].

The well-established evidence that various EPH/ephrin members exhibit tumor-promoting features has challenged researchers to develop therapeutic interventions that target the EPH/ephrin signaling pathway, as shown in Table 3. Searching the existing literature, we found that plenty of therapeutic strategies were suggested, such as pazopanib and trametinib in osteosarcoma [19], ALW-II-41-27 in osteosarcoma, Ewing’s sarcoma and chondrosarcoma [20], dasatinib against ARMS [29], trichostatin A (TSA) and suberoylanilide hydroxamic acid (SAHA) in ERMS [35], VasG3 and sEPHB4-HAS in ERMS [36], GLPG1790 in combination with radiation therapy in ERMS [37], and the design of EPHB4-CAR-T cells against osteosarcoma and rhabdomyosarcoma [38]. On the contrary, several antibodies that target molecular members of the EPH/ephrin axis have been already tested in other solid tumors in clinical trials: for instance, the anti-EPHA2 monoclonal antibody DS-8895a in gastric and esophageal cancer patients, the anti-EPHA3 antibody IIIA4 (Ifabotuzumab/KB004) in glioblastoma patients and anti-ephrin-A4-Calicheamicin (PF-06647263) in breast and ovarian cancer patients with promising results [44]. Overall, more in vitro and in vivo research studies were carried out evaluating the key role of the EPH/ephrin signaling pathway in soft tissue sarcomas, especially for rhabdomyosarcoma, compared to bone tissue sarcomas. However, we observed an absence of clinical studies evaluating the addition of therapeutic agents targeting the EPH/ephrin signaling pathway and its mediators for bone and soft tissue sarcomas.

## 6. Conclusions

In the present review of the literature, we highlight multiple mechanisms implicating the EPH/ephrin signaling pathway in bone (osteosarcoma, chondrosarcoma, Ewing’s sarcoma) and soft tissue sarcomas (synovial sarcoma, rhabdomyosarcoma) based on published in vitro and in vivo studies. Mostly, the upregulation of EPH receptors leads to tumor-promoting functions in bone sarcomas such as tumor cells migration and proliferation by EPHA2, along with enhanced carcinogenesis and invasion by EPHA7 in osteosarcoma. Likewise, EPHA2 induces genetic mutations in chondrosarcoma and amplifies tumor growth, migration and angiogenesis in Ewing’s sarcoma. Similarly, these oncogenic effects are demonstrated in soft tissue sarcomas when EPHA3, EPHB2 and EPHB4 stimulate properties such as proliferation, increased motility, migration, invasion and metastasis of rhabdomyosarcoma and synovial sarcoma cells. There is also increasing evidence that ephrin ligands contribute to the progression of bone and soft tissue sarcomas. For instance, ephrin-B1 enables invasive characteristics and resistance against chemotherapy of osteosarcoma cells and induces the migratory features of ERMS cells. Importantly, a tumor-suppressive effect was noticed by ephrin-A5 in chondrosarcoma due to an exclusive inhibitory effect against carcinogenesis and tumor progression. Therefore, based on our literature review, ephrin-A5 is the only molecule from the EPH/ephrin axis that could have a protective anti-cancer role in musculoskeletal sarcomas.

Understanding the activities of EPH/ephrin axes in bone and soft tissue sarcomas is of paramount importance to design novel therapeutics for the treatment of local and the prevention of metastatic disease. Despite our efforts to combine data from laboratory and clinical trials, which are indicative of the suppressive or promoting effect of the EPH/ephrin system, we found ephrin-B1 as the only common molecule from the EPH/ephrin axis being studied among bone and soft tissue sarcomas. According to our analysis, ephrin-B1 is responsible for the increased invasiveness of ERMS cells and poorer prognosis of ERMS compared to ARMS patients. Similarly, ephrin-Bi enhances proliferation, migration, invasion and chemoresistance in osteosarcoma. To our view, activities of EPH/ephrin axes are similar in bone and soft tissue sarcomas, as most of the molecules involved induce cancerous processes, except ephrin-A5. However, there are a lack of data to prove that the same action is promoted by a specific EPH or an ephrin either in bone or in soft tissue sarcomas. At the present time, we strongly believe that we have to consider each pathology in regard to already conducted studies on the EPH/ephrin system’s participation until future ones will clarify whether common EPHs or ephrins play the same role in bone and soft tissue sarcomas.

The significant impact of EPHs and ephrins in musculoskeletal sarcoma’s development and progression indicates that these molecules could represent promising monitoring tools as well as theranostic biomarkers and a new direction for ad hoc chemotherapeutic drug design. Treatment options targeting the EPH/ephrin axis have been evaluated in vitro and in vivo with encouraging outcomes. We have noticed that anti-EPHA2 and anti-EPHB4 as well as anti-ephrin-B1 therapeutic agents (Table 3) have been explored in bone and soft tissue sarcomas, albeit they could have also been tried in the clinical setting.

Finding the most significant signaling pathway that implicates the EPH/ephrin system is crucial for the investigation of new therapeutics against bone and soft tissue sarcomas. To our knowledge, the unregulated PI3K/AKT or RAS/MAPK pathways exhibit a central role in the progression of musculoskeletal sarcomas, and they are able to trigger a plethora of well-defined cancerous cascades [45]. From our perspective, EPHAs and their co-expressed ligands, ephrin-As, constitute the best mediators for the PI3K/AKT and RAS/MAPK pathways. Therefore, we believe that the EPHA/ephrin-A axis should be prioritized as the most promising and effective therapeutic target.

Based on our literature review, we have distinguished the study by Fritsche-Guenther et al. [15] as the most representative one about the diagnostic value of EPHA2 and ephrin-A1 for the early detection of osteosarcoma. Interestingly, the de novo expression of EPHA2 in clinical osteosarcoma specimens and the proportional increase during progression from conventional to metastatic stages upgrades EPHA2 to a remarkable diagnostic biomarker. Similarly, the significant upregulation of ephrin-B and EPHB in RMS has validated them as potential diagnostic biomolecules for the progression of soft tissue sarcomas [27].

However, the absence of clinical studies investigating the application of EPH/ephrin target therapies was noticed in recent literature. Consequently, we consider the design of clinical studies that will further elucidate the key role of EPHs and ephrins as potential therapeutic targets for the suppression of bone and soft tissue sarcomas and the prevention of end-stage metastatic disease of paramount importance.

## Figures and Tables

**Table 1:** Molecular mechanisms involving the EPH/ephrin axis in bone sarcomas leading to specific laboratory and clinicopathological outcomes.

EPH/Ephrin	Tumor Type	Cell Lines/Tissues	Mechanism	Result/Clinicopathological Correlations	References
EPHA2	OS	8 OS cell lines: SaOS2, HOS, MNNG⁄HOS, OST, SJSA, MG63, ZK58 19 OS tissue samples: 7/19 metastatic OS 12/19 conventional OS Control: Primary osteoblast cells (HOBc) Fetal and normal adult tissue samples	De novo expression of EPHA2 Activation of Ras/MAPK signaling pathway Suppression of Fos and Jun (downstream effectors)	EPHA2/ephrin-A1 interaction induces: excessive proliferation and migration of OS cells ↑ EPHA2 → tumor development and metastatic disease	[12]
	EWS	In vitro: A673, TC252, RH1 and STAET1 cell lines In vivo: knocked down Caveolin-1 (CAV1) expression in RDES, TC71 and SKES1 cells of EWS xenograft mice Control: Non-transfected cells and cells transfected with an empty vector	EPHA2/ephrin-A1 has a CAV1-dependent interaction that activates AKT signaling ↑ transcription of bFGF	↑ EPHA2-dependent activity promotes EWS angiogenesis EPHA2-CAV1 axis → endothelial cell migration ↓ primary tumor growth in CAV1 knocked down xenografts (associated with EPHA2 expression) No significant changes in the malignancy of EWS cells transfected with the EPHA2 dominant-negative construct	[13]
	OS	7 OS cell lines: EPHA2-silenced osteosarcoma cell lines (HOS, KHOS/NP, MNNG/HOS and U2OS) vs. Controls OS xenograft mice MNNG/HOS or KHOS Application or not of RTK pathway’s inhibitors (pazopanib + trametinib)	pazopanib + trametinib down-modulated the expression of EPHA2 and IL7R and upregulated MEK6 expression	EPHA2-silenced OS and pazopanib + trametinib treated cells: ↓ cell viability inhibition of cell migration	[16]
	OS EWS CHS	EPHA2 expression retrieved using Bioinformatics Analyses Patients(n): 232 OS, 197 EWS, 102 CHS Cell lines: 10 OS, 12 EWS, 4 CHS Patient-Derived Xenograft Models (PDX models) Osteoblastic OS (metastatic) EWS (localized) CHS (conventional)	↑ expression of EPHA2 in the following bone sarcoma cell lines: (Saos-2, U2OS, MG63) followed by EWS cell lines (SK-NEP-1, RD-ES, CADO-ES1) and CHS cell lines (CAL-78, Hs 819.T, SW 1353) Phosphorylation of serine 897 in EPHA2 (*p*-EPHA2*^Ser897^*) Activates oncogenesis in OS and CHS PDX models	OS patients: ↑ expression of EPHA2 in tumors with a higher Huvos grade No significant association with survival outcomes ↑ expression to males compared to females EWS patients: ↑ expression of EPHA2 in tumor samples compared to normal tissue Significant upregulation to males compared to females CHS patients: ↑ levels of EPHA2 correlated with worse prognosis in dedifferentiated CHS Significant association between mutational status of CHS and EPHA2 expression PDX models: EPHA2 inhibitor ALW II-41-27 reduced cell viability and tumor growth in OS, EWS and CHS	[17]
EPHA7	OS	Tissue samples from 40 OS patients	↑ HCP5 expression OS tissues compared to control ↑ HCP5 expression in OS cell lines (MG-63, U2OS, 143B, and HOS) compared with normal cells (hFOB1.19) Knockdown of HCP5 suppressed cell proliferation, migration, invasion and enhanced cell apoptosis in MG-63 and U2OS cells HCP5 regulates the expression of miR-101 by targeting miR-101 in OS miR-101 directly targets the 3′UTR region of EPHA7 and mediates the EPHA7 expression in OS cell lines	HCP5 expression is enhanced in OS cell lines and tissues The HCP5/miR-101/EPHA7 axis is involved in OS development HCP5 induces OS cell proliferation, migration, and invasion by up-regulation of EPHA7 (targeting miR-101 competitively)	[19]
		OS cell lines: MG-63, U2OS, 143B, HOS, human osteoblast cell line (hFOB)			
ephrin-B1	OS	Tissues from 12 OS patients vs. controls OS cell lines: U2OS, MG63,143B OS xenografts mice: OS cells transfected with the NICD1-OE, RBPJ-shRNA vs. control	Activation of Notch signaling → phosphorylation of ephrin-B1 and increases the expression of ephrin-B1 Inhibition of Notch signaling is able to reduce tumor growth and metastasis in xenografts mice. Notch intracellular domain (NICD) is transferred to the nucleus and activates the transcriptional cofactor CBF1, leading to the overexpression of *HES* and *HEY* genes and ephrin-B1 in 143B OS cell lines	Notch signaling promotes proliferation, migration, invasion, upregulation of stem-cell like abilities and chemoresistance by targeting ephrin-B1	[14,15]
ephrin-A5	CHS	19 patients: 15 conventional CHS 6 CHS grade I 9 CHS grade II 4 dedifferentiated CHS vs. 3 patients normal articular cartilage Cell lines: Human CHS cell lines C3842 and SW1353	Not identified mechanism of ephrin-A5 downregulation in CHS. No significant differences in the expression of ephrin-A5 in C3842 and SW1353 cells treated with or without hypoxia. *Ephrin-A5 gene* promoter hypermethylation is not the cause of *ephrin-A5 gene* downregulation in CHSs.	Protective function in tumor progression ↓ ephrin-A5 leads to tumorigenesis, tumor progression and ↓ cellular adhesion	[18]

Abbreviations: OS: osteosarcoma; EWS: Ewing’s sarcoma; CHS: chondrosarcoma; CAV: caveolin; PDX: patient-derived xenografts; RTK: receptor tyrosine kinase; HCP5: histocompatibility leukocyte antigen (HLA) complex P5 (HCP5); NICD: Notch intracellular domain.

**Table 2:** Molecular mechanisms involving the EPH/ephrin axis in soft tissue sarcomas leading to specific laboratory and clinicopathological outcomes.

EPH/Ephrin	Soft Tissue Sarcoma	Cell Lines/Tissues	Mechanism	Result/Clinicopathological Correlations	References
EPHB2	SS	NIH3T3 cells infected with either SYT-SSX2 cDNA vs. control retroviral pOZ backbone	SYT-SSX2 → increased expression and activation of the EPHB2	EPHB2-mediated cytoskeletal remodeling: Elongation and narrowing of SS cells → neurite-like extensions Loss of cell–cell adhesions Stimulation of the metastatic cascade	[22]
EPHA3	RMS	Cell lines: 3 ARMS cell lines (KM77, CRL2061, FLOH-1) and 2 ERMS lines (RD and TE671)	Upregulation of EPHA3 in two ERMS (TE671 and RD) and one ARMS (FLOH-1) cell lines that do not express the PAX3-FKHR fused oncogene ↓ levels of EPHA3 in highly aggressive ARMS cell lines (CRL2061 and KM77) that exhibit the PAX3-FKHR fused oncogene ephrin-A5/Fc and EPHA3 interaction regulates RMS cell adhesion + migration (modulation of Rho GTPases)	Tumor-suppressive effect of EPHA3/ephrin-A5 in RMS. Ligation of EPHA3 by ephrin-A5/Fc stimulation caused decreased cell adhesion to fibronectin and decreased migration toward SDF-I	[25]
	ERMS	Cell lines: TE671/WT and TE671/RAGE cells	TE671/RAGE cells downregulated EPHA3, A4, A5 and B2 and ephrin-A1, A3, A4 and B3 + Upregulated EPHA7 and ephrin-B2, with EPHA3 and ephrin-A1 showing the greatest differential expression compared to TE671/WT cells EPHA3 and ephrin-A1 upregulated after forced expression of PAX7 in TE671/RAGE cells ↓ neural cell adhesion molecule (NCAM1) in ERMS cells.	PAX7-overexpressing cells → excessive migration and invasion (upregulation of EPHA3)	[28]
EPHB4	ARMS	Human skeletal muscle and ARMS cell lines (Rh5, Rh30, Rh3, Rh18) Murine ARMS cell lines (U23674 and U48484)	PDGF-BB ligand promotes direct activation of PDGFRβ and indirect activation of EPHB4 Activation of Akt and Erk1/2 signaling pathways.	↑ ARMS cell’s survival and proliferation. EPHB4 overexpression → poor overall survival in PAX3:FOXO1 positive ARMS patients.	[26]
ephrin-B1	ERMS and ARMS	Cell lines: GFP-expressing control cells vs. RD cells overexpressing ephrin-B1 Human study: Comparison of ephrin-B1 expression levels between 102 ERMS patients and 130 ARMS patients	HDACs regulates the expression of ephrin-B1	↑ migration of ERMS cells in vitro ephrin-B1 expression is differentially increased in ERMS patients compared to ARMS patients Poorer survival of ERMS compared to ARMS patients.	[30]

Abbreviations: SS: synovial sarcoma; RMS: rhabdomyosarcoma; ARMS: alveolar rhabdomyosarcoma; ERMS: embryonic rhabdomyosarcoma; NCAM: neural cell adhesion molecule; PDGF: platelet-derived growth factor; HDACs: histone deacetylases.

**Table 3:** Chemotherapeutic agents targeting the EPH/ephrin axis that have been proposed for the treatment of bone and soft tissue sarcomas.

EPH/Ephrin Target	Bone/Soft Tissue Sarcoma	Chemotherapeutic Agent	Mechanism	Result/Clinicopathological Correlations	References
EPHA2	OS	Pazopanib and Trametinib	Down-modulation of RTK EPHA2 and Interleukin-7 Receptor (IL-7R) Inhibition of ERK1/2 and Akt Induces mitogen-activated protein-kinase kinase (MAPKK) MEK6.	↓ proliferation and migration of OS cells Synergistic anti-tumor effectiveness	[16]
	OS EWS CHS	ALW-II-41-27	Direct EPHA2 inhibitor	↓ cell growth and cell viability	[17]
	ARMS	Dasatinib	Antagonistic effect against PDGFRβ (PDGFRβ /PDGFR-BB axis) and EPHB4 (EPH/ephrin axis) Inhibition of the Src family kinases	Inhibition of expansion of ARMS cells in vitro and prolonged survival of ARMS xenografts in vivo	[26]
EPHB4	ARMS and ERMS OS	VasG3 antibody serum EPHB4-HSA (human serum albumin)	Direct inhibition of EPHB4	sEPHB4-HAS slightly decreased tumor growth in ARMS murine models VasG3 and sEPHB4-HAS could not be used as monotherapy treatments	[31]
	ARMS OS	Engineered chimeric antigen receptor (CAR)-T cells	Direct inhibition of EPHB4	In vitro: Reduced proliferation of ARMS cells ↑ necrosis of OS and ARMS cells In vivo: ↑ survival of ARMS xenografts mice ↓ growth rates of primary tumors	[33]
EPHA2	ERMS	GLPG1790	pan-EPH inhibitor In vitro: phosphorylation/activation of EPHA2 and EPHBs and inhibited activation of AKT/mTOR and MEK/ERK axis Enhanced JNKs-mediated apoptosis Occluded p-38 sustained differentiation Deteriorated motility and invasion features by restricting SRC-mediated integrin signals Enhanced radiosensitivity of ERMS cells by impairment of the DNA double-strand break repair	↓ proliferation and reversed malignant phenotype of ERMS cells In vivo: Combination of GLPG1790 *+* radiation therapy Up to 83% reduction in primary tumor growth in ERMS xenograft models	[32]
ephrin-B1	ERMS	Trichostatin A (TSA) Vorinostat: Suberoylanilide hydroxamic acid (SAHA)	In vivo: HDAC inhibitors Enriched binding of acetylated histones on the e*phrin-B1* promoter region	↓ migration of ERMS cells	[30]

## Data Availability

The data presented in this study are available on request from the corresponding author.

## Abbreviations

Eph	erythropoietin-producing hepatocellular carcinoma receptors
CAV	caveolin protein
RMS	rhabdomyosarcoma
ERMS	embryonal rhabdomyosarcoma
ARMS	alveolar rhabdomyosarcoma
miR	micro-RNA
HDACs	histone deacetylases
CAR	chimeric antigen receptor
